# Validating a composite endpoint for acceptability evaluation of oral drug formulations in the pediatric population: a randomized, open-label, single dose, cross-over study

**DOI:** 10.3389/fphar.2024.1436554

**Published:** 2024-08-19

**Authors:** Juliane Münch, Anna Lena Schwarzwälder, Carolin Kloft, Hans Martin Bosse, Manfred Wargenau, Sibylle Reidemeister, Ingrid Klingmann, Viviane Klingmann

**Affiliations:** ^1^ Department of General Pediatrics, Neonatology and Pediatric Cardiology, Medical Faculty and University Hospital Duesseldorf, Heinrich Heine University, Duesseldorf, Germany; ^2^ M.A.R.C.O. GmbH & Co. KG, Institute for Clinical Research and Statistics, Duesseldorf, Germany; ^3^ Novartis Pharma AG, Global Drug Development/Technical Research and Development, Novartis Campus, Basel, Switzerland; ^4^ Pharmaplex Bv, Wezembeek-Oppem, Belgium

**Keywords:** acceptability, palatability, oral pediatric formulations, swallowability, drug administration

## Abstract

**Objective:**

This study aimed to validate the newly developed composite acceptability endpoint to investigate acceptability of oral pediatric drug formulations that integrates swallowability and palatability assessments.

**Methods:**

In this open-label study acceptability of oral formulations was tested in three age groups (1–<6 months, 6–<12 years, and 12–<18 years) with a 2-way cross-over design in children aged 1–<6 months (syrup and mini-tablets), and with an incomplete block design of four sequences with three out of four formulations (syrup, mini-tablets, oblong tablet, and round tablet) each in children aged 6–<18 years. The primary endpoint was acceptability derived from the composite acceptability endpoint. Secondary endpoints were palatability and acceptability derived from swallowability.

**Results:**

A total of 320 children were stratified into three age groups (80 children aged 1–<6 months, 120 children aged 6–<12 years, and 120 children aged 12–<18 years). All participants completed the study. Age-specific differences were observed in acceptability derived from the composite acceptability endpoint. Mini-tablets had the highest acceptability in participants aged 1–<6 months and 6–<12 years while the oblong tablet was leading in adolescent participants (12–<18 years).

**Conclusion:**

This study demonstrated that the composite acceptability endpoint method integrating both swallowability and palatability assessments is a sensitive method to assess acceptability of drug formulations in children of different age.

**Clinical Trial Registration:**

https://drks.de/search/de, identifier DRKS00027948.

## 1 Introduction

Appropriate formulations in pharmacotherapy for children are a crucial factor for patients’ adherence to the prescribed treatment. The current practice of administrating liquids or syrup in children results in a surprisingly unreliable dosing with substantial under- or over-dosage (Yin et al., 2009). Thus, it is not only necessary to investigate the efficacy and optimal doses of pharmaceutical substances for different pediatric age groups but also to develop adapted galenic formulations for the most suitable routes of administration.

A variety of pediatric formulations such as solutions, suspensions, mini-tablets of different diameters, oblong tablets and orodispersible films have been developed. Although many different investigational methods have been applied to assess acceptability, swallowability, and palatability of such formulations, at present there is no validated method to assess acceptability in a standardized, comprehensive way that includes both, the swallowing process and the palatability reactions that would allow comparing and prioritizing the acceptability of different oral formulations.

In previous studies Klingmann et al. investigated the swallowability of different oral pediatric formulations in children between 2 days and 6 years in addition to palatability (Klingmann et al., 2020; Münch et al., 2021). The swallowability was observed and assessed by a trained investigator. The administration was video filmed, and palatability was evaluated by two independent blinded raters in a standardized setting. To further increase the capability to reliably distinguish the acceptability of oral formulations for different age groups a composite acceptability endpoint was developed that integrates assessment results of both swallowability and palatability in the same participant. This newly defined composite acceptability endpoint method was shown to be able to better discriminate between four tested oral formulations than the previous definition, which was based on swallowability only (Wargenau et al., 2022).

The overall aim of this study was to validate this composite acceptability endpoint method for acceptability evaluation (combination of palatability and swallowability scores) of four oral drug formulations in a pediatric population of different age groups.

## 2 Participants and methods

### 2.1 Study participants

The pediatric participants (registered as inpatients or outpatients) were recruited in the Department of General Pediatrics, Neonatology and Pediatric Cardiology of the University Hospital Düsseldorf, Germany, between 09 August 2022, and 09 December 2022. A total of 320 evaluable children, stratified into three age groups (80 children aged 1–<6 months, 120 children aged 6–<12 years, and 120 children aged 12–<18 years), were included. Apart from the age and given informed consent the main inclusion criteria was the ability to swallow. Children with impaired swallowing ability, interrupted regular oral feeding after an operation, lactose-intolerance, medication-caused nausea, fatigue, or palsy, or sickness after eating food before the study were excluded. The detailed inclusion and exclusion criteria of the study participants are shown in Table 1.

**TABLE 1:** Eligibility criteria.

Inclusion criteria	Exclusion criteria
Male or female participants aged from 1 to <6 months and from 6 to <18 years	Impairment of swallowing solids due to illness
Ability to swallow	Lactose-intolerance
Willingness and capability of participants and participants’ parents or legal guardians to comply with examination procedures	Premedication and concomitant medication that caused nausea, fatigue, or palsy
Participants and/or participants’ parents or legal guardians capable of providing written informed consent and assent where possible	Children in the post-operative period who were yet to commence regular oral feeding
	Children, who had eaten 1 h before examination and who afterwards felt sick because of the food

### 2.2 Formulations

The following oral formulations were used: glucose syrup manufactured by Caesar & Loretz GmbH; uncoated age-adapted number of mini-tablets with a diameter of 2 mm, age-adapted sized uncoated round tablets with a diameter of 6 mm (age group 2) or 13 mm (age group 3), and age-adapted sized uncoated oblong tablets with a dimension of 6 × 2.5 mm (age group 2) or 14.5 × 5.7 mm (age group 3). All tablet formulations were manufactured by NextPharma. All four formulations were drug-free. Tablet formulations consisted of lactose, cellulose, magnesium stearate, and anhydrous colloidal silicon dioxide. Glucose syrup contained glucose and water.

### 2.3 Study design

**FIGURE 1:**
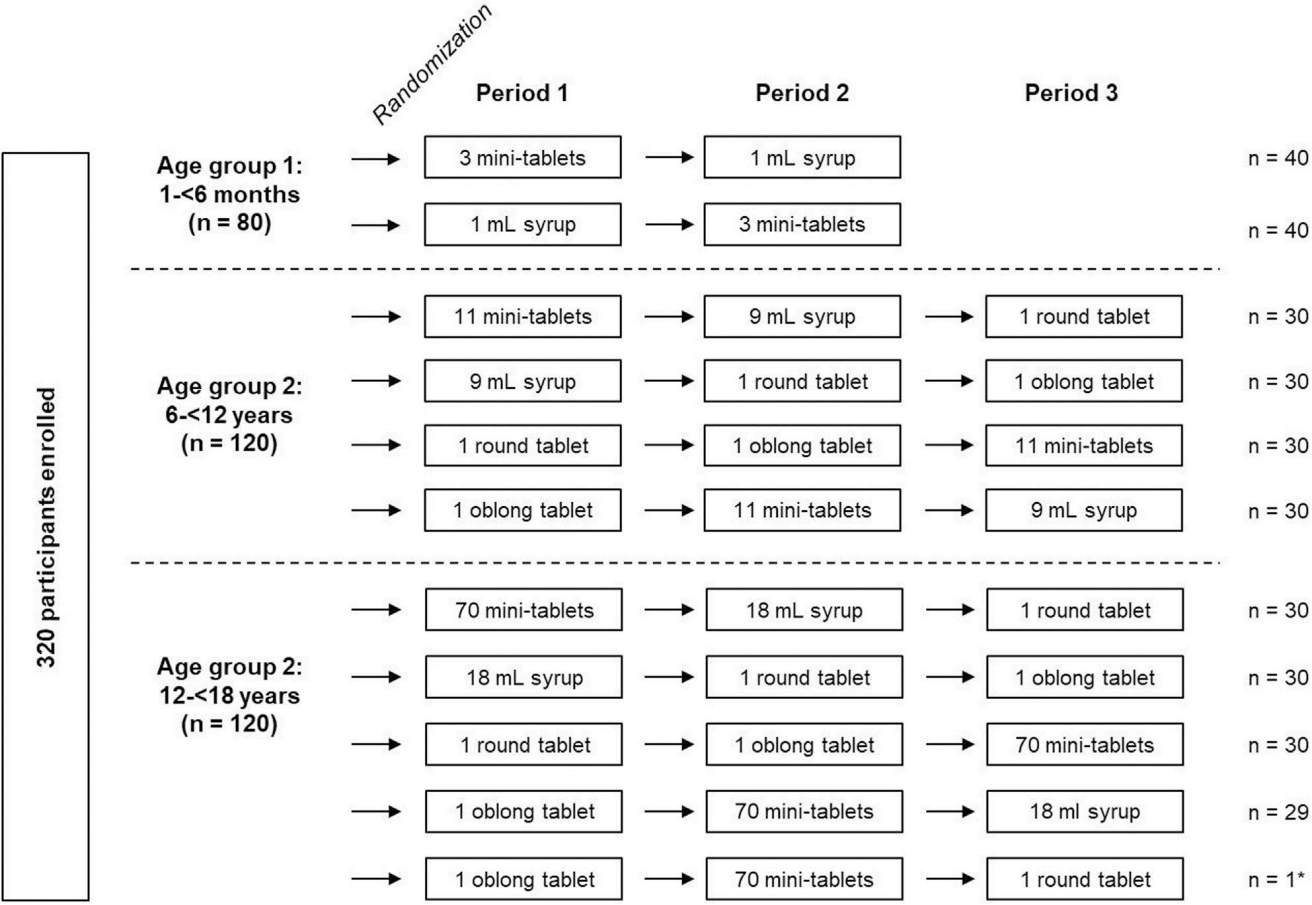
Study design and randomization of participants *1 participant received a non-planned cross-over sequence. Data were used for analysis.

The study design is shown in Figure 1. This open-label study was performed in three age groups (age group 1: 1–<6 months; age group 2: 6–<12 years, and age group 3: 12–<18 years) with a 2-way cross-over design in children aged 1–<6 months and with an incomplete block design of four sequences with three out of four formulations each in children aged 6–<18 years. Since relevant formulations (i.e., mini-tablets and orodispersible film) have been investigated for children aged 6 months to 6 years in previous studies (Spomer et al., 2012; Klingmann et al., 2013, 2015, 2018, 2020; Münch et al., 2021) and the calculation of the composite acceptability endpoint has been based on two of these studies (Wargenau et al., 2022), this age group was not included in this study.

#### 2.3.1 Randomization

Randomization was performed stratifying by age groups using a self-developed and validated SAS^®^ macro RANDOM which is based on the SAS^®^ function RANUNI for uniformly distributed variables. Within the age group 1–<6 months, children were randomized to one of two treatment sequences (mini-tablets/syrup; syrup/mini-tablets). In the other two age groups (6–<12 years and 12–<18 years) each participant was randomized to one of four sequences in an incomplete block cross-over design to receive three out of four formulations (multiple mini-tablets, glucose syrup, round and oblong tablet) in randomized order (see Figure 1).

#### 2.3.2 Administration procedure

For formulation administration the participant and (if wished by the participant) the parent(s) were seated in a quiet, distraction-free area. The different formulations were taken by mouth according to the randomization scheme. The mini-tablets, round and oblong tablets were placed on the tongue with a teaspoon. The participant had to swallow the tablet formulations with water or a drink at his/her choice. Alternatively, the tablets were placed on a teaspoon together with a soft food (if age permitted) and administered to the participant. The syrup was administered with a syringe in all age groups. The swallowing process and the child’s reactions were thoroughly observed and documented by the specially trained investigator. 45 s after placing the formulation into the child’s mouth, the mouth was inspected by the investigator. Palatability was video documented and assessed by the investigator and a second independent rater in the age group 1–<6 months and in the two older age groups as self-assessment by a 5-point Likert scale with smileys 30 s after swallowing.

In the second and third study period, the processes were repeated with the other formulation(s) on the same day as the first period.

#### 2.3.3 Endpoints

The primary endpoint was defined as acceptability derived from the composite acceptability endpoint and expressed as binary outcome (“yes,” “no”). Secondary endpoints were palatability assessment expressed as binary outcome and acceptability derived from swallowability. Adverse events (AEs) and swallowing problems were also monitored and reported.

#### 2.3.4 Evaluation criteria

**FIGURE 2:**
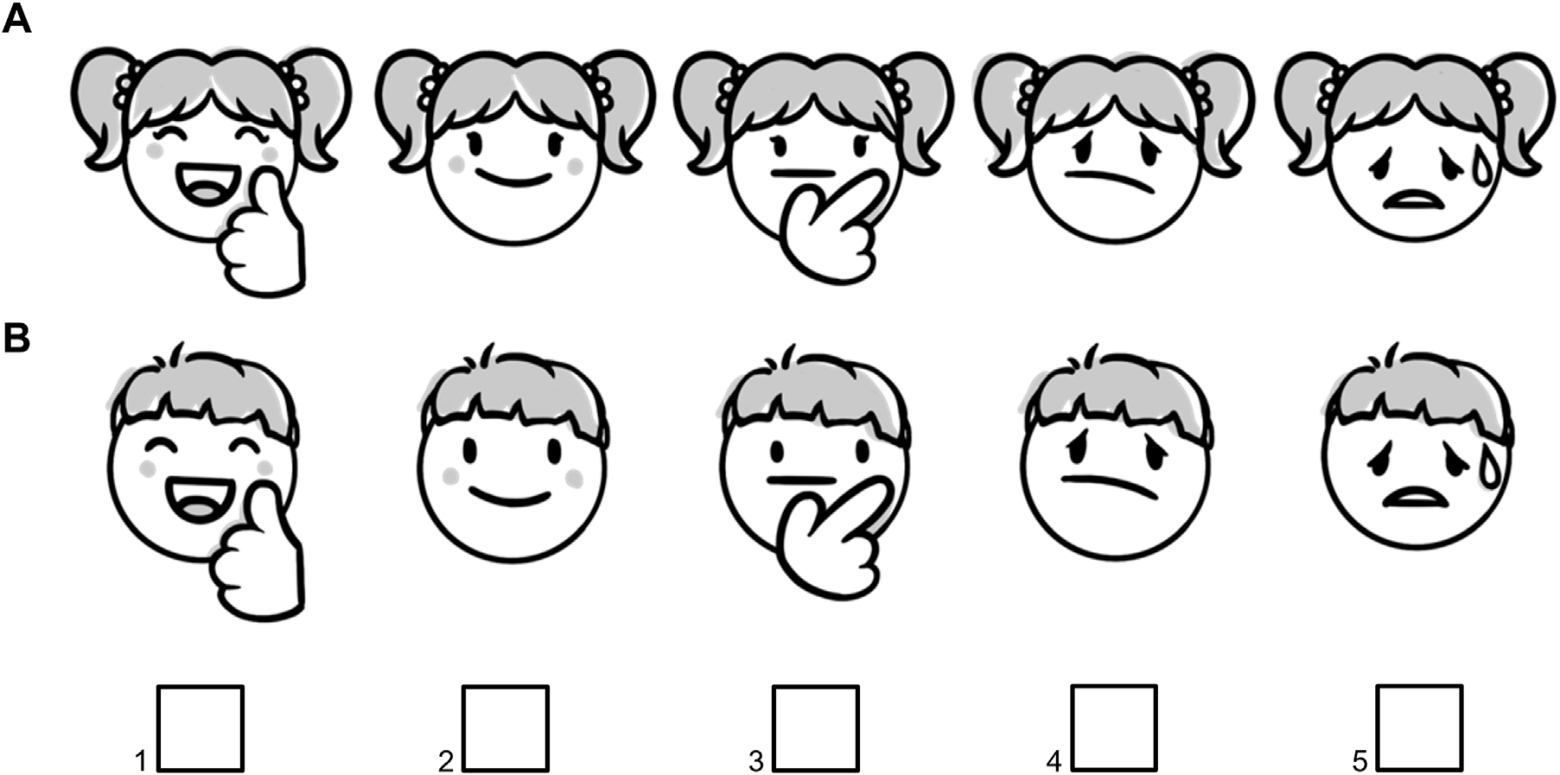
Sex-adapted 5-point Likert scale for palatability self-assessment in participants aged 6–<18 years for female **(A)** and male **(B)** participants. Scoring system: very pleasant = 1, pleasant = 2, neutral = 3, unpleasant = 4, very unpleasant = 5.

Swallowability and palatability were assessed separately with formulation- and age-adapted scoring methods as shown in Table 2 (swallowability assessment), Table 3 (palatability assessment in participants aged 1–<6 months) and Figure 2 (palatability assessment in participants aged 6–<18 years). Thereby, the observer/rater described the swallowing process physiologically connected to a numbering system as “Swallowed = 1,” “Chewed/left over = 2,” “Spat out = 3,” “Swallowed the wrong way/coughed = 4” and “Refused to take = 5.” Palatability was judged as “pleasant,” “neutral” or “unpleasant” depending on the facial reaction of the child or via self-assessment. Acceptability was defined as either based on swallowability assessment only with a swallowability score 1–2 defining “acceptable” whereas “not acceptable” was connected to a swallowability score >2 (Table 4) or based on the new combination of swallowability and palatability assessment (composite acceptability endpoint, Table 5). For example, a swallowability score of 1 and a palatability rating of “pleasant” were summarized to “high” acceptability.

**TABLE 2:** Scoring criteria for swallowability for tablet formulations (A) and syrup (B).

A: Scoring criteria for swallowability: tablet formulations		
Score	Observation	
1	Swallowed	No chewing took place during swallowing and no residuals of the solid were found during oral inspection
2	Chewed/left over	Chewing was observed before swallowing and/or that the whole or parts of the solid were found in the mouth during oral inspection and/or left over on the spoon. ≥80% should be swallowed
3	Spat out	No swallowing took place and the solid was no longer in the child’s mouth
4	Swallowed the wrong way/coughed	The solid was swallowed the wrong way or a cough was caused
5	Refused to take	The child didn’t allow the investigator to place the solid in the mouth

B: Scoring criteria for swallowability: syrup		
Score	Observation	
1	Swallowed	No residuals of the syrup were found during oral inspection
2	Chewed/left over	The whole or parts of the syrup were found in the mouth during oral inspection and/or left over in the syringe. ≥80% should be swallowed
3	Spat out	No swallowing took place and the syrup was no longer in the child’s mouth
4	Choked on	The syrup was swallowed the wrong way or a cough was caused
5	Refused to take	The child didn’t allow the investigator to place the syrup in the mouth

**TABLE 3:** Scoring criteria for palatability based on video documentation per rater (A) and combined (B) for participants aged 1–<6 months.

A: Scoring criteria for palatability per rater		
Score	Observation	
1	Pleasant	Positive hedonic pattern: Tongue protrusion, smack of mouth and lips, finger sucking, corner elevation
2	Neutral	Neutral mouth and body movements, and face expression
3	Unpleasant	Negative aversive pattern: Gape, nose wrinkle, eye squinch, frown, grimace, head shake, arm flail

B: Scoring for palatability combined			
Scoring of rater no. 1	Scoring of rater no. 2		
	Pleasant	Neutral	Unpleasant
Pleasant	Pleasant	Pleasant	Contradictory
Neutral	Pleasant	Neutral	Unpleasant
Unpleasant	Contradictory	Unpleasant	Unpleasant

**TABLE 4:** Definition of acceptability derived from swallowability alone as binary outcome.

	Criteria	
Acceptable	Yes	Swallowability score was 1 or 2
	No	Swallowability score was >2 (3–5)

**TABLE 5:** Definition of acceptability derived from the composite acceptability endpoint.

A: Definition of composite acceptability endpoint as a function of swallowability and combined palatability			
Palatability	Swallowability score		
	1	2	≥3
Very pleasant/pleasant	High	Good	No
Neutral	Good	Low	No
Very unpleasant/unpleasant	Low	No	No
Contradictory	Good	Low	No

B: Composite acceptability endpoint expressed as binary outcome			
	Criteria		
Acceptable	Yes	Composite acceptability endpoint “high” or “good”	
	No	Composite acceptability endpoint “low” or “no”	

#### 2.3.5 Determination of sample size

For the 2 × 2 cross-over design a sample size of 72 participants was regarded as appropriate. Based on this sample size, a difference of at least 15%-points (e.g., 90% vs. 75%) could be detected with 80% power at a significance level α = 10% (2-sided). 80 children were planned to be enrolled and randomized in the age group 1–<6 months to take account for dropouts. In the two older age groups, pairwise comparisons between 4 formulations were performed in an incomplete block design. Therefore, 120 participants were planned to be enrolled and randomized in each of these age groups.

#### 2.3.6 Statistical analyses

All statistical calculations were carried out using SAS^®^ language and procedures (SAS^®^ 9.4 version, SAS-Institute, Cary NC, United States). The imputation of missing data was not performed.

Demographic data and baseline characteristics were presented descriptively by age group, sequence group, and overall. Categorical data were summarized by frequencies and percentages, continuous data by number of observations, means, standard deviation, minimum, first quartile, median, third quartile, and maximum. Frequency tables (including counts and percentages) were provided for the primary endpoint (acceptability: no/yes) secondary endpoints (all outcomes of swallowability and all outcomes of palatability) by age group and formulation. Moreover, 2 × 2 contingency tables were provided presenting paired outcomes (i.e., result after one formulation vs. another) by age group and overall.

In age group 1–<6 months, the primary endpoint of acceptability was analyzed as binary outcome according to the cross-over design. Acceptability rates were compared between the two formulations by applying the analysis proposed by Schouten and Kester (Schouten and Kester, 2010). At first, the difference in acceptability rates between the two formulations was estimated for each sequence group and then averaged over both sequence groups in a second step. Corresponding 2-sided 90% CIs were calculated for the averaged difference of acceptability rates.

In the two older age groups, formulations were compared in pairwise manner based on 2 × 2 contingency tables applying the McNemar test. Two-sided 90% CIs were calculated for all pairwise differences of acceptability rates.

Potential effects of age group and formulation on the study endpoint and respective interaction were investigated by application of categorical data analysis with repeated measures using log-linear modelling of the frequencies presented in a contingency Table 1. This analysis was carried out by using the SAS procedure CATMOD.

Analyses of secondary endpoints were performed analogously. All analyses were performed on the full analysis set.

## 3 Results

### 3.1 Disposition of participants

A total of 320 participants were enrolled: 80 in age group 1 (1–<6 months) with 40 participants per cross-over sequence, and 120 in age group 2 (6–<12 years) and age group 3 (12–<18 years) each with 30 participants per cross-over sequence. In age group 3, one participant received a non-planned sequence (oblong tablet/mini-tablets/round tablet instead of oblong tablet/mini-tablets/syrup). Data from this participant were still used for analysis. All 320 participants completed the study (Figure 1).

### 3.2 Primary endpoint

**FIGURE 3:**
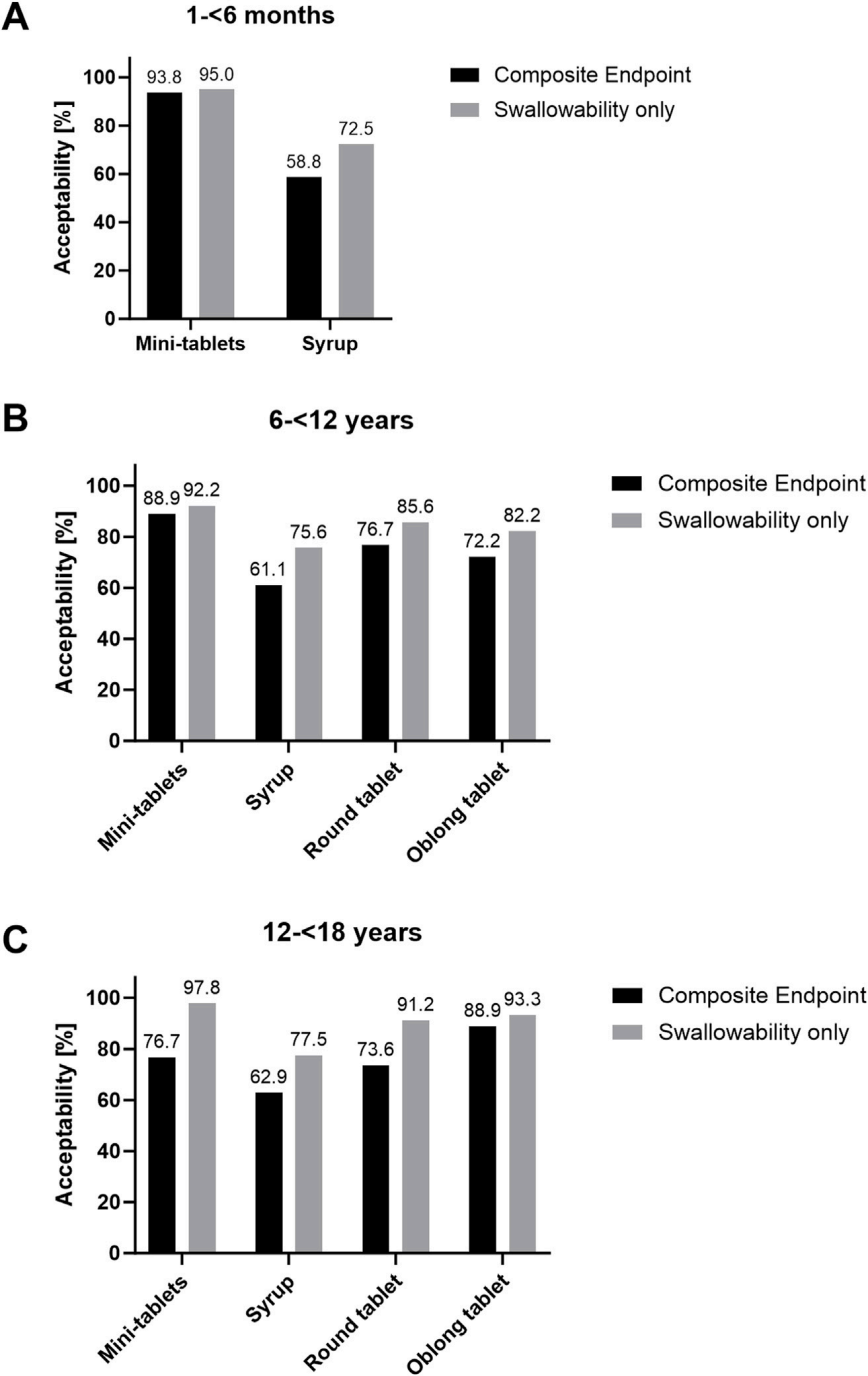
Acceptability rate of oral formulations: Black bars: Acceptability assessment via composite endpoint from the combination of palatability and swallowability. Grey bars: Acceptability assessment based on swallowability only. N = 80 for age group 1 and N = 120 for age group 2 and 3.

Acceptability based on the composite acceptability endpoint assessment was high for mini-tablets in age group 1 while acceptability of syrup was lower (Figure 3A). The difference in the acceptability rate of 35.0% over both cross-over sequences for comparison between mini-tablets and syrup was significant (90% CI: 23.4%; 46.6%; *p* < 0.0001) (Supplementary Table S1).

In age group 2, the highest acceptability rate was observed for mini-tablets, followed by round tablet and oblong tablet. Syrup had the lowest acceptability rate in children aged 6–12 years (Figure 3B). The difference in the acceptability rate for mini-tablets compared to syrup (+25.0%; 90% CI: +13.7%; +36.3; *p* = 0.0003), to round tablet (+15.0%; 90% CI: +5.1%; +24.9%; *p* = 0.0126) and to oblong tablet (+18.3%; 90% CI: +5.8%; +30.9%; *p* = 0.0164), respectively, was significant. The acceptability rate for syrup was significantly lower compared to round tablet (−18.3%; 90% CI: −30.3%; −6.4%; *p* = 0.0116) and oblong tablet (−20.0%; 90% CI: −32.9%; −7.1%; *p* = 0.0105). No statistically significant difference was observed for acceptability rates of round versus oblong tablet in this age group (Supplementary Table S2).

In age group 3, the highest acceptability rate was observed for oblong tablets, followed by mini-tablets and round tablets. Syrup had the lowest acceptability rate in adolescents aged 12–18 years (Figure 3C). The difference in acceptability rate for mini-tablets (−15.0%; 90% CI: −26.3%; −3.7%; *p* = 0.0290), syrup (−23.7%; 90% CI: −37.9%; −9.5%; *p* = 0.0060) as well as round tablet (−18.0%; 90% CI: −28.5%; −7.6%; *p* = 0.0045) compared to oblong tablet was significant. The acceptability rate for mini-tablets was significantly higher compared to syrup (+18.6%; 90% CI: +4.7%; +32.6%; *p* = 0.0278). The comparison of the acceptability rates for mini-tablets vs. round tablet and syrup vs. round tablet revealed no statistically significant differences in this age group (Supplementary Table S3).

Potential effects of age group and formulation on the primary study endpoint and respective interaction were investigated and results are summarized in Table 6. Since there were significant age-specific results concerning some comparisons of formulations, age and formulation effects were not interpretable in some comparisons. For example, in age group 2 (6–<12 years) mini-tablets showed a higher acceptability rate (88.9%) than the oblong tablet (72.2%) while the outcome was the opposite in age group 3 (12–<18 years), namely 76.7% vs. 88.9%, respectively).

**TABLE 6:** Results of testing age and formulation effects on the composite acceptability endpoint (*p*-values are provided).

Comparison	Age	Formulation	Interaction age-by-formulation
Mini-tablets vs. syrup	0.5985	<0.0001	0.2256
Mini-tablets vs. round tablet	Not interpretable	Not interpretable	0.0863
Mini-tablets vs. oblong tablet	Not interpretable	Not interpretable	0.0007
Round tablet vs. syrup	0.3325	0.0050	0.6580
Oblong tablet vs. syrup	0.0242	<0.0001	0.7331
Oblong tablet vs. round tablet	Not interpretable	Not interpretable	0.0300

In case of a significant age-by-formulation interaction (*p* < 0.1) overall age and overall formulation results are not interpretable

Mini-tablets showed a higher acceptability compared to syrup consistently over all three age groups. The round tablet showed a consistently higher acceptability compared to syrup for the two older age groups.

Acceptability rates of mini-tablets vs. round tablets were not consistent for the two older age groups as indicated by the *p*-value < 0.1 for the interaction term. The acceptability rate for the mini-tablets was significantly higher compared to the round tablet in the age group 2 (6–<12 years, *p* = 0.0126), whereas no difference between the two formulations was evident in the age group 3 (12–<18 years, *p* = 0.8348). A similar pattern was observed for the comparison of mini-tablets vs. oblong tablet, but with a clearly more pronounced age-specific effect (as indicated by the *p*-value < 0.001 for the interaction term).

The acceptability rate for the mini-tablets was significantly higher compared to the oblong tablet in the age group 2 (6–<12 years, *p* = 0.0164), whereas it was significantly lower in the age group 3 (12–<18 years, *p* = 0.0290). The oblong tablet showed a consistently higher acceptability compared to syrup for the two older age groups with significant outcomes in both groups. Furthermore, a significant difference between the two age groups was detected: The acceptability rate was higher for each of the two formulations in the age group 3 (12–<18 years) compared to age group 2 (6–<12 years) leading to an overall difference of about 15% between age groups.

With regard to the comparison of the oblong vs. the round tablet, the results were not consistent for the two older age groups as indicated by the *p*-value of 0.03 for the interaction term: In fact, the acceptability rate for oblong tablet was significantly higher compared to the round tablet in the age group 12–<18 years (*p* = 0.0045), whereas no difference between the two formulations was evident in the age group 6–<12 years (*p* = 0.3173).

### 3.3 Secondary endpoints

#### 3.3.1 Acceptability based on swallowability

Acceptability based only on swallowability assessment was high for mini-tablets in participants aged 1–<6 months (age group 1) and lower for syrup (Figure 3A). The difference in acceptability rates of 13.8% for mini-tablets vs. syrup over both cross-over sequences was significant (90% CI: 5.8%; 21.7%; *p* = 0.0045) (Supplementary Table S4). In age group 2, the acceptability rate for mini-tablets was higher compared to syrup (+15.0%; 90% CI: 5.1%; 24.9%; *p* = 0.0126), oblong tablet (+13.3%; 90% CI: 3.1%; 23.6%; *p* = 0.0325), and round tablet (+10.0%; 90% CI: 2.2%; 17.8%; *p* = 0.0339) in this age group. The acceptability rate for syrup was lower compared to round (−15.0%; 90% CI: −24.9%; −5.1%; *p* = 0.0126) and oblong tablet (−13.3%; 90% CI: −23.6%; −3.1%; *p* = 0.0325). No statistically significant difference in acceptability was observed for round vs. oblong tablet in participants aged 6–12 years (Figure 3B and Supplementary Table S5).

In age group 3, the acceptability rate for mini-tablets was higher compared to syrup (+22.0%; 90% CI: 12.0%; 32.1%; *p* = 0.0003). The acceptability rate for syrup was lower compared to oblong tablet (−17.0%; 90% CI: −28.8%; −5.1%; *p* = 0.0184). No statistically significant difference in acceptability was observed for other comparisons of formulations in participants aged 12–18 years (Figure 3C and Supplementary Table S6).

Potential effects of age group and formulation on the secondary study endpoint acceptability derived from swallowability and respective interaction were investigated and results summarized in Table 7.

**TABLE 7:** Results of testing age and formulation effects on acceptability derived from swallowability (*p*-values are provided).

Comparison	Age	Formulation	Interaction age-by-formulation
Mini-tablets vs syrup^a^	—	—	—
Mini-tablets vs round tablet	0.1808	0.0173	0.2283
Mini-tablets vs oblong tablet	0.0068	0.0149	0.1440
Round tablet vs syrup	1.0000	0.0034	0.7142
Oblong tablet vs syrup	0.3513	0.0009	0.6910
Oblong tablet vs round tablet	0.0784	0.0996	0.9891

^a^ No results given because the statistical model could not be fitted due to multi-collinearity. Since the acceptability rate for mini-tablets based on swallowability was 100% in the age group 3 there were no cases in the response categories ‘mini-tablet not acceptable / syrup not acceptable’ and ‘mini-tablet not acceptable / syrup acceptable’ (Supplementary Table S6).

Regarding the comparison of mini-tablets vs. round tablets, a statistically significant difference can be recognized (*p* = 0.0173) with an estimated overall difference of 6.6% in favor of the mini-tablet (10.0% in age group 2, and 3.3% in age group 2). Significant differences between formulations were also detected concerning mini-tablets vs. oblong tablet with an overall difference of 8.3% in favor of the mini-tablet, as well as for round tablet vs. syrup and oblong tablet vs. syrup both to the disadvantage of syrup. Since none of the interaction terms was statistically significant, no age-specific effects concerning the comparative acceptability of drug formulations could be detected by the swallowability approach.

#### 3.3.2 Palatability assessment

**FIGURE 4:**
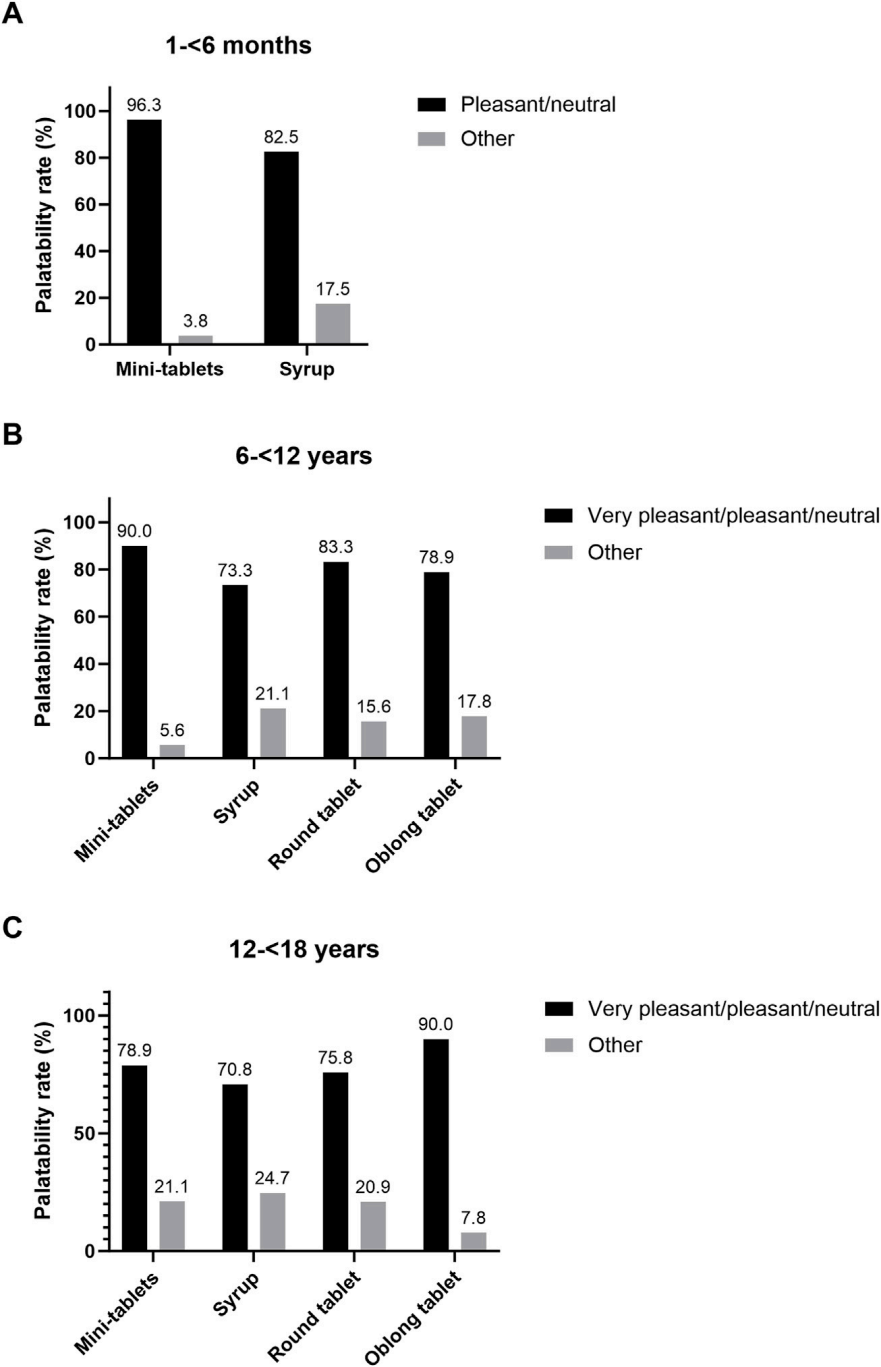
Palatability rate as binary outcome. **(A)** Age group 1 (N = 80), combined assessment of two investigators and in **(B)** age group 2 and **(C)** age group 3 (N = 120 each) self-assessment by participants was used.

In age group 1, palatability was assessed by video documentation and rating by two independent investigators. Based on a 3-scoring criteria system, “neutral” was the most common outcome for both mini-tablets (87.5%) and syrup (60.0%). Overall, a higher palatability rate was observed for mini-tablets in this age group compared to syrup (Figure 4A). In the two older age groups, self-assessment of the palatability was performed. In age group 2, mini-tablets were most often assessed as palatable, followed by round tablet, oblong tablet, and syrup. (Figure 4B). In age group 3, the highest rate of ‘very pleasant/pleasant/neutral’ was assessed for oblong tablet, followed by mini-tablets, round tablet, and syrup (Figure 4C).

### 3.4 Safety assessment

No AEs were reported in this study. Swallowing problems were only reported in age group 1 (1–<6 months) in three out of 80 administrations of mini-tablets. For these participants, the swallowability criterion 4 “swallowed the wrong way/coughed” was documented. None of these events were of clinical relevance and were not assessed as AEs.

## 4 Discussion

In this study, the acceptability of oral formulations was assessed by a newly developed composite acceptability endpoint integrating swallowability and palatability (primary endpoint) (Wargenau et al., 2022) and by the previously used approach which was solely based on swallowability (secondary endpoint).

In all age groups, acceptability rates based on the composite acceptability endpoint were consistently lower for all investigated drug formulations than those which only accounted for swallowability. This, of course, was expected since palatability had been added as relevant component of acceptability in the new approach.

In several cases incorporation of palatability had a considerable impact on the assessment of acceptability. For syrup, acceptability assessed by the composite acceptability endpoint was reduced in all age groups compared to acceptability based on swallowability only (58.8% vs. 72.5% in age group 1, 61.1% vs. 75.6% in age group 2, and 62.9% vs. 77.5% in age group 3).

The most prominent difference between the two acceptability definitions was observed in age group 3 (12–<18 years). Integration of palatability led to a distinctly lower rate of acceptability for mini-tablets (76.7% vs. 97.8% when solely based on swallowability).

Method validation was performed in different pediatric age groups with placebo formulations due to ethical reasons. Therefore, specific properties of an active drug formulation as taste or smell, which may affect palatability and consequently acceptability, could not be considered. Thus, validation had to be carried out under presumably unfavorable conditions, i.e., with placebo formulations having a neutral taste and smell. Furthermore, due to the study design, blinding for the participants and rater was not possible. Nevertheless, it was shown that the new acceptability approach was highly suitable to discriminate between different formulation principles.

Although swallowability and palatability are two important qualifiers for acceptability, the authors are aware that the acceptability concept as outlined by the European Medicines Agency (EMA) guideline (EMA Guideline, 2013) comprises more characteristics such as handling of the medicinal product, also refer to the EMA letter of support for the presented composite endpoint method (EMA Support Letter, 2023).

Furthermore, the composite acceptability endpoint method was even found to be sufficiently sensitive to detect age-specific effects, in particular with regard to the comparative acceptability of drug formulations. For example, the acceptability rate was significantly higher for the oblong tablet compared to the round tablet in the age group 3 (12–<18 years), whereas no difference between the two formulations was apparent in the age group 2 (6–<12 years) leading to a significant age group-by–formulation interaction. Furthermore, in age group 2 (6–<12 years) mini-tablets showed a higher acceptability rate (88.9%) than the oblong tablet (72.2%) while the outcome was the opposite in age group 3 (12–<18 years), namely, 76.7% vs. 88.9%, respectively.

This validation study also demonstrated that the applied scoring systems assessing swallowability and palatability are appropriate. In particular, all single scores of the 5-point Likert scale concerning palatability assessments were observed for each investigated placebo formulation in the age groups ‘6–<12 years’ and ‘12–<18 years’ (i.e., ‘very pleasant’, ‘pleasant’, ‘neutral’, ‘unpleasant’, ‘very unpleasant’).

In summary, this study demonstrated that the composite acceptability endpoint method integrating both swallowability and palatability assessment is a sensitive method to compare acceptability of drug formulations in pediatric participants of different age.

## Data Availability

The raw data the conclusions of this article will be made available by the authors, without undue reservation.
