# Supplementary material for: Validating a composite endpoint for acceptability evaluation of oral drug formulations in the pediatric population: a randomized, open-label, single dose, cross-over study
Source: Front Pharmacol. 2024 Aug 19;15:1436554. doi: 10.3389/fphar.2024.1436554 (PMC11366573; doi:10.3389/fphar.2024.1436554)
Supplement: Supplementary file 1 [file Table1.DOCX]

Supplementary Material

## Supplementary Tables

**Table S1** Comparison of acceptability rates based on composite acceptability endpoint in age group 1 (1-<6 months)

| Arm | N | Mini-tablets [%] | Syrup [%] | Rate difference [%] | 90% CI | | p-value |
| --- | --- | --- | --- | --- | --- | --- | --- |
|  |  |  |  |  | Lower | Upper |  |
| Sequence 1  (Age Group 1) | 40 | 95.0 | 62.5 | 32.5 | 24.5 | 40.5 | <.0001 |
| Sequence 2  (Age Group 1) | 40 | 92.5 | 55.0 | 37.5 | 29.0 | 46.0 | <.0001 |
| All | 40 | 93.8 | 58.8 | 35.0 | 23.4 | 46.6 | <.0001 |
| CI = confidence interval, FAS = full analysis set, N = number of participants | | | | | | | |

**Table S2** Comparison of acceptability rates based on composite acceptability endpoint in age group 2 (6-<12 years)

| Comparison | | N | P(T) [%] | P(R) [%] | Rate difference [%] | 90% CI | | p-value |
| --- | --- | --- | --- | --- | --- | --- | --- | --- |
| Test (T) | Reference (R) |  |  |  |  | Lower | Upper |  |
| Mini-tablets | Syrup | 60 | 90.0 | 65.0 | 25.0 | 13.7 | 36.3 | 0.0003 |
| Mini-tablets | Round tablet | 60 | 91.7 | 76.7 | 15.0 | 5.1 | 24.9 | 0.0126 |
| Mini-tablets | Oblong tablet | 60 | 85.0 | 66.7 | 18.3 | 5.8 | 30.9 | 0.0164 |
| Syrup | Round tablet | 60 | 63.3 | 81.7 | -18.3 | -30.3 | -6.4 | 0.0116 |
| Syrup | Oblong tablet | 60 | 55.0 | 75.0 | -20.0 | -32.9 | -7.1 | 0.0105 |
| Round tablet | Oblong tablet | 60 | 71.7 | 75.0 | -3.3 | -8.8 | 2.1 | 0.3173 |
| CI = confidence interval, FAS = full analysis set, N = number of participants, P = probability | | | | | | | | |

**Table S3** Comparison of acceptability rates based on composite acceptability endpoint in age group 3, (12-<18 years)

| Comparison | | N | P(T) [%] | P(R) [%] | Rate difference [%] | 90% CI | | p-value |
| --- | --- | --- | --- | --- | --- | --- | --- | --- |
| Test (T) | Reference (R) |  |  |  |  | Lower | Upper |  |
| Mini-tablets | Syrup | 59 | 81.4 | 62.7 | 18.6 | 4.7 | 32.6 | 0.0278 |
| Mini-tablets | Round tablet | 61 | 75.4 | 77.0 | -1.6 | -14.6 | 11.3 | 0.8348 |
| Mini-tablets | Oblong tablet | 60 | 73.3 | 88.3 | -15.0 | -26.3 | -3.7 | 0.0290 |
| Syrup | Round tablet | 60 | 60.0 | 73.3 | -13.3 | -28.3 | 1.7 | 0.1441 |
| Syrup | Oblong tablet | 59 | 66.1 | 89.8 | -23.7 | -37.9 | -9.5 | 0.0060 |
| Round tablet | Oblong tablet | 61 | 70.5 | 88.5 | -18.0 | -28.5 | -7.6 | 0.0045 |
| CI = confidence interval, FAS = full analysis set, N = number of participants | | | | | | | | |

**Table S4** Comparison of acceptability rates based on swallowability in age group 1 (1-<6 months)

| Arm | N | Mini-tablets [%] | Syrup [%] | Rate difference [%] | 90% CI | | p-value |
| --- | --- | --- | --- | --- | --- | --- | --- |
|  |  |  |  |  | Lower | Upper |  |
| Sequence 1  (Age Group 1) | 40 | 95.0 | 82.5 | 12.5 | 7.1 | 17.9 | 0.0002 |
| Sequence 2  (Age Group 1) | 40 | 97.5 | 82.5 | 15.0 | 9.2 | 20.8 | <.0001 |
| All | 40 | 96.3 | 82.5 | 13.8 | 5.8 | 21.7 | 0.0045 |
| CI = confidence interval, FAS= full analysis set, N = number of participants | | | | | | | |

**Table S5** Comparison of acceptability rates based on swallowability in age group 2 (6-<12 years)

| Comparison | | N | P(T) [%] | P(R) [%] | Rate difference [%] | 90% CI | | p-value |
| --- | --- | --- | --- | --- | --- | --- | --- | --- |
| Test (T) | Reference (R) |  |  |  |  | Lower | Upper |  |
| Mini-tablets | Syrup | 60 | 91.7 | 76.7 | 15.0 | 5.1 | 24.9 | 0.0126 |
| Mini-tablets | Round tablet | 60 | 95.0 | 85.0 | 10.0 | 2.2 | 17.8 | 0.0339 |
| Mini-tablets | Oblong tablet | 60 | 90.0 | 76.7 | 13.3 | 3.1 | 23.6 | 0.0325 |
| Syrup | Round tablet | 60 | 76.7 | 91.7 | -15.0 | -24.9 | -5.1 | 0.0126 |
| Syrup | Oblong tablet | 60 | 73.3 | 86.7 | -13.3 | -23.6 | -3.1 | 0.0325 |
| Round tablet | Oblong tablet | 60 | 80.0 | 83.3 | -3.3 | -8.8 | 2.1 | 0.3173 |
| CI = confidence interval, FAS = full analysis set, N = number of participants | | | | | | | | |

**Table S6** Comparison of acceptability rates based on swallowability in age group 3 (12-<18 years)

| Comparison | | N | P(T) [%] | P(R) [%] | Rate difference [%] | 90% CI | | p-value |
| --- | --- | --- | --- | --- | --- | --- | --- | --- |
| Test (T) | Reference (R) |  |  |  |  | Lower | Upper |  |
| Mini-tablets | Syrup | 59 | 100.0 | 78.0 | 22.0 | 12.0 | 32.1 | 0.0003 |
| Mini-tablets | Round tablet | 61 | 96.7 | 93.4 | 3.3 | -2.1 | 8.7 | 0.3173 |
| Mini-tablets | Oblong tablet | 60 | 96.7 | 93.3 | 3.3 | -2.1 | 8.8 | 0.3173 |
| Syrup | Round tablet | 60 | 78.3 | 90.0 | -11.7 | -23.6 | 0.3 | 0.1083 |
| Syrup | Oblong tablet | 59 | 76.3 | 93.2 | -17.0 | -28.8 | -5.1 | 0.0184 |
| Round tablet | Oblong tablet | 60 | 90.2 | 93.4 | -3.3 | -7.1 | 0.5 | 0.1573 |
| Source: Table 14.2.2-4  CI = confidence interval, FAS = full analysis set, N = number of participants | | | | | | | | |
